# Adipose-derived mesenchymal stem cells (ASCs) may favour breast cancer recurrence via HGF/c-Met signaling

**DOI:** 10.18632/oncotarget.1359

**Published:** 2013-10-23

**Authors:** Vincenzo Eterno, Alberto Zambelli, Lorenzo Pavesi, Laura Villani, Vittorio Zanini, Gianfranco Petrolo, Stefania Manera, Antonella Tuscano, Angela Amato

**Affiliations:** ^1^ Laboratory of Experimental Oncology and Pharmacogenomics, IRCCS Salvatore Maugeri Foundation, Pavia; ^2^ Unit of Medical Oncology, IRCCS Salvatore Maugeri Foundation, Pavia; ^3^ Breast Unit, IRCCS Salvatore Maugeri Foundation, Pavia; ^4^ Unit of Pathology, IRCCS Salvatore Maugeri Foundation, Pavia

**Keywords:** Adipose-derived Mesenchymal Stem Cells (ASCs), Breast Cancer, HGF/c-Met crosstalk, Microenvironment, Neoangiogenesis

## Abstract

Adipose tissue is a reservoir of Mesenchymal Stem Cells (Adipose-derived Mesenchymal Stem Cells, ASCs), endowed with regenerative properties. Fat graft was proposed for breast reconstruction in post-surgery cancer patients achieving good aesthetic results and tissues regeneration. However, recent findings highlight a potential tumorigenic role that ASCs may have in cancer recurrence, raising some concerns about their safety in clinical application.

To address this issue, we established a model where autologous ASCs were combined with primary normal or cancer cells from breast of human donors, in order to evaluate potential effects of their interactions, in vitro and in vivo.

Surprisingly, we found that ASCs are not tumorigenic per sè, as they are not able to induce a neoplastic transformation of normal mammary cells, however they could exhacerbate tumorigenic behaviour of c-Met-expressing breast cancer cells, creating an inflammatory microenvironment which sustained tumor growth and angiogenesis.

Pharmacological c-Met inhibition showed that a HGF/c-Met crosstalk between ASCs and breast cancer cells enhanced tumor cells migration, acquiring a metastatic signature, and sustained tumor self-renewal.

The master role of HGF/c-Met pathway in cancer recurrence was further confirmed by c-Met immunostaining in primary breast cancer from human donors, revealing a strong positivity in patients displaying a recurrent pathology after fat grafts and a weak/moderate staining in patients without signs of recurrence.

Altogether our findings, for the first time, suggest c-Met expression, as predictive to evaluate risk of cancer recurrence after autologous fat graft in post-surgery breast cancer patients, increasing the safety of fat graft in clinical application.

## INTRODUCTION

Autologous fat graft is used as a filler for breast reconstruction in cancer patients following conservative surgery. Previous reports showed the regenerative capability of Adipose-derived Mesenchymal Stem Cells (ASCs) in several medical fields such as plastic, orthopedic, cardiac, oral/maxillofacial and breast surgery. Autolougous fat grafts were used to correct irregularities and, surprisingly they showed to promote a local tissue repair as a result of a new microenvironment, where ASCs favour healing processes [1], as it was shown for tissue damaged by radiotherapy in post-surgery breast cancer patients [2].

Recently, it was found that mesenchymal stem cells are essential for proper tissues development and homeoastasis as described for mammary gland [3], however little is known about mechanisms of interactions between mesenchymal stem cells and breast cancer cells in tumor microenvironment, namely, if mesenchymal stem cells may favour epithelial growth toward a tumorigenic development, or vice versa, if breast cancer cells could influence mesenchymal stem cells dictating a tumor-supporting behaviour.

Mesenchymal stem cells are well known to secrete cytokines, chemokines and growth factors essential for development and maintenance of an inflammatory state, improving physiological tissue regeneration after injury.

However, it was found that a well orchestrated inflammatory response supports tumorigenesis, creating an optimal microenvironment where cancer cells are continuously stimulated to proliferate, also by recruitment of several cell types able to promote tumor neoangiogenesis [4, 5]. Besides, inflammation contributes to metastasis favouring homing of disseminated tumor cells in new tissues [6]. This is feasible because breast tumor cells also produce cytokines and growth factors, and express their cognate receptors which could be activated both in a paracrine and autocrine fashion [7].

Several cytokines, chemokines and growth factors were found to mediate a crosstalk between epithelial cells and surrounding stromal cells, which could reveal determinant in cancer initiation, progression and metastatic spread [8-10]. Cytokines, such as SDF-1, support proliferation and migration of breast cancer cells expressing CXCR4 receptor [11], as well as high serum levels of Interleukin-6 and Interleukin-8 are associated with poor outcome in breast cancer patients [12, 13].

Some growth factors produced and released in tumor microenvironment, such as PDGF and VEGF, were shown to take part in tumor neoangiogenesis promoting differentiation of recruited endothelial progenitors into new vessels [14, 15].

Moreover, it was found that mesenchymal stem cells support tumorigenesis also influencing breast cancer phenotype, in terms of aggressiveness and invasive capability, such as supporting Epithelial-Mesenchymal Transition (EMT) which was reported to precede tumor cell dissemination and metastasis [16-19]. The ability of microenvironment to influence tumor phenotype was also found in a mouse model of accelerated host aging (Cav-1^−/−^), where it was shown that mammary tumorigenesis is favoured by a senescent microenvironment defined by the loss of stromal Cav-1 expression, in a fashion that is estrogen- and progesterone-independent [20].

Besides, epithelium-stroma crosstalk was found to maintain the small sub-population of cancer cells (Cancer-Initiating Cells, CICs), present as quiescent cells in the bulk of a tumor, characterized by multi-drugs resistance and self-renewal, likely responsible for recurrence, acquired chemoresistance and metastatic spread [21-25].

Altogether those findings reveal a pleiotropic effect of mesenchymal stem cells in regulating CICs, tumor proliferation, migration and angiogenesis; therefore tumor stroma is a heterogenic microenvironment where different cells reside and communicate with each other via a complex signaling network. Consequently, the development of therapies, targeting both tumor cells and mesenchymal stem cells, may reveal a more effective strategy in treating cancer [26-28].

Based on these evidences, poor understanding of Adipose-derived Mesenchymal Stem Cells (ASCs) biological properties compromises their safety in clinical application [29, 30].

Indeed, those findings discourage the employment of ASCs for regenerative/reconstructive purpose in cancer patients, what is further supported by an expanding body of literature with several clinical reports showing a fast-growing and metastatic recurrence after fat graft in cancer patients, following tumor eradication [31, 32].

In particular, although ASCs regenerative properties demonstrated to achieve good aesthetic results improving the health of the tissues in the setting of fat graft-mediated breast reconstruction in post-surgery breast cancer patients, however, if residual cancer cells persist following breast-conserving surgery, ASCs could be a promoter of cancer recurrence, likely exacerbating aggressiveness and metastatic spread of breast cancer cells, which would make more difficult the management of recurrence itself.

So far no molecular marker was suggested as predictive of recurrence, what would help surgeons to choose among patients who really will benefits of fat graft and in which cases it would be risky. Moreover, previously published works investigated the role of ASCs in tumorigenesis employing established human cell lines from breast cancer, however little is known about interaction between ASCs and primary breast cancer cells [33], isolated from primary breast tumor [34], which could depict a situation closer to reality.

Our work shed light on the role that ASCs could have in breast cancer recurrence of post-surgery patients undergoing autologous fat graft, dissecting signaling pathways which could sustain a crosstalk between primary breast cancer cells and ASCs favouring tumorigenesis.

We established a model where autologous ASCs and primary breast cancer cells from human donors were combined, in order to evaluate potential effects of their interactions, in vitro and in vivo.

Our findings showed that ASCs are not tumorigenic per sè, however they reveal a tumorigenic potential in presence of c-Met-expressing breast cancer cells.

A crosstalk mediated by HGF/c-Met between ASCs and cancer cells, stimulates acquisition of highly invasive capabilities of cancer cells, which increased their growth rate and self-renewal potential, supported by beta-catenin activation. In this scenario, ASCs-produced cytokines and chemokines creates a microenvironment supporting neo-angiogenesis and inflammation.

Those findings suggest c-Met as a predictive marker to evaluate risk of recurrence after fat graft in post-surgery breast cancer patients, where residual cancer cells could still persist.

Our work proposes a molecular explanation for those clinical cases reporting a fast-growing, more aggressive recurrence after fat graft, besides increases the safety of fat graft in clinical applications, suggesting c-Met as a molecular marker driving surgeons to identify which patients would be at major risk of recurrence.

## RESULTS

### ASCs isolated from lipoaspirates of human donors express mesenchymal and stem markers and display multipotency

Adipose tissue is considered a source of adult stem cells of mesenchymal origins [35]. Those cells share several features with haematopoietic mesenchymal stem cells, however they display proper identity.

Previously, a method to isolate mesenchymal stem cells from lipoaspirates of adipose tissue was reported [1]. Accordingly with that report we processed lipoaspirates from human donors, isolating the stromal vascular fraction which was further evaluated for content in ASCs.

**Figure 1:**
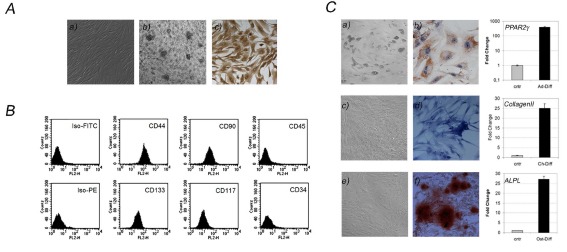
Lipoaspirate: a source of ASCs A) Fibroblast-like morphology (a), adhesion-independent growth (b) and vimentin expression (c) evaluated in ASCs isolated from human lipoaspirates. B) Evaluation of mesenchymal, stem (CD44, CD90, CD133, CD117), haematopoietic (CD45) and endothelial markers (CD34). C) ASCs differentiation into adipocytes (a, morphologic analysis and b, Oil Red-O staining) chondrocytes (c, morphologic analysis and d Alcian Blue Staining) and osteocytes (e, morphologic analysis and f, Alizarin Red staining), expressing specific genes of mature cells. Data are results of three independent experiments, each considering a triplicate of each sample.

Isolated cells displayed a fibroblast-like morphology (Figure 1A, a) and adhesion-independent growth as well, since they formed foci growing in matrigel (Figure 1A, b). In addition those cells expressed vimentin (Figure 1A, c), a well known mesenchymal marker.

Cytofluorimetry revealed the expression of surface markers supporting their stemness and mesenchymal features as CD44, CD133, CD117, and CD90, in contrast low percentage of CD34 positive cells were found suggesting a low presence of endothelial progenitors in isolated cells populations. In addition, no CD45 positive subpopulation was found, indicating isolated cells were not of haematopoietic origin (Figure 1B).

To evaluate multipotency of ASCs, we induced differentiation in vitro toward the adipogenic, chondrogenic or osteogenic lineages using different culture media, each containing lineage-specific factors. We obtained, with high efficiency, mature adipocytes (Figure 1C, a) containing lipid drops inside their cytoplasms, confirmed by Oil-Red-O staining (Figure 1C, b). In addition, Q-PCR revealed those cells displaying an increased expression of PPAR2-gamma, a gene specifically expressed in mature adipocytes (Figure 1C, upper graph). Chondrocytes differentiation was assessed by morphology analysis (Figure 1C, c) and Alcian Blue staining (Figure 1C, d), detecting the presence of a collagen matrix produced by chondrocytes, which expressed increased levels of collagen II gene (Figure 1C, middle graph). Then, Alizarin Red assessed osteocytes differentiation (Figure 1C, f) staining calcium phosphates produced by mature osteocytes (Figure 1C, e), which revealed to express high levels of ALPL gene, a well known enzime of bone tissue (Figure 1C, bottom graph).

Those findings, aimed to characterize cells population isolated from lipoaspirates, revealed those cells respected minimal criteria to be defined ASCs [36].

### ASCs influence proliferation and migratory capabilities of some primary breast cancer cells in vitro

To investigate whether isolated ASCs could influence primary breast cancer cells behaviour in vitro, we established a transwell assay allowing us to perform indirect co-culture of both mesenchymal and epithelial breast cancer cells.

In this study, we isolated ASCs and primary breast cancer cells from human lipoaspirates or breast cancer tissues, respectively, from the same human donor and combined them in order to establish a model, in vitro or in vivo, to evaluate potential effects of their interactions.

Four Ductal Invasive Carcinoma, characterized by histological markers as hormonal receptor status (ER, PR, Her2), Ki67, grade (Additional file, Table 1) were processed to isolate primary breast cancer cells (indicated as KBr1, KBr2, KBr3, KBr4). Evaluation of luminal and basal markers, cytokeratin 8 (CK8) and vimentin (Vmt) respectively as well as E-cadherin (E-cad) and beta-catenin (β-cat) localization revealed that those cells exhibited a luminal-like phenotype, further confirmed by EpCam expression evaluated by cytofluorimetry (Additional file, Figure 1).

Proliferation assay revealed an increased proliferation rate (about 30% for KBr1, KBr2, KBr3, and 15% for KBr4 at three days) for breast cancer cells when co-cultured with autologous ASCs (ASC1, ASC2, ASC3 and ASC4, respectively), in comparison with breast cancer cells cultured alone (Additional file, Figure 2A). A lower increase (about 10%) in proliferation rate was found in normal epithelial cells isolated from normal mammary gland of each human donor (Additional file, Figure 3B), characterized by morphological analysis and expression of breast markers (EpCam, E-cadherin and cytokeratin 8, Additional file, Figure 3A).

**Figure 2:**
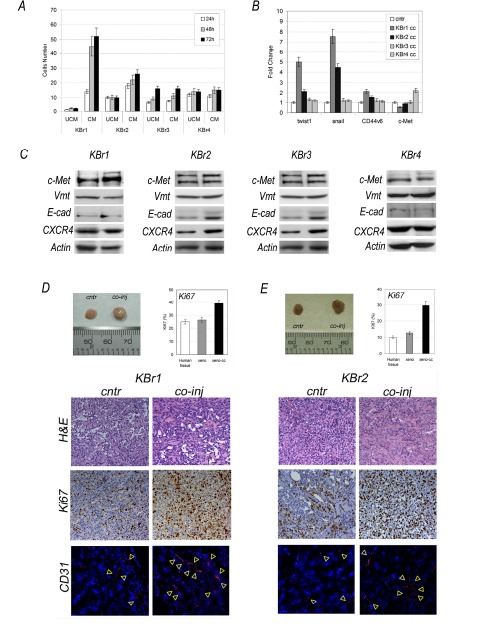
ASCs are not tumorigenic per sè A) Migratory activity, B) Metastatic signature and C) c-Met, vmt, E-cad and CXCR4 protein levels in co-cultured KBr cells (cc) versus KBr cells (cntr); for migratory activity ASCs-conditioned (CM) versus unconditioned (UCM) medium. D) and E) Xenografts, Ki67 evaluation, H&E and CD31 staining (red, yellow-arrowhead, nuclei counterstained with DAPI) in KBr1 and KBr2, respectively, injected into nude mice with ASCs (co-inj) or alone (cntr).

In order to evaluate if ASCs were able to influence migratory activity of breast cancer cells in vitro, we performed a transwell migration assay (Figure 2A). We found that two out four samples increased their migratory activity in presence of ASCs-conditioned medium (KBr1 and KBr2). The remaining two samples (KBr3 and KBr4), even if showed migratory capacity *per sè*, however it was not exacerbated by ASCs. In contrast, ASCs-conditioned medium failed to chemoattract normal epithelial cells which did not show any migration potential.

Increased migratory capabilities in KBr1 and KBr2 cells, suggested they could have acquired a metastatic signature, defined by expression of specific genes as twist1, snail1, frequently overexpressed in cancer cells with high metastatic potential [37, 38], as well as c-MET receptor and its co-receptor CD44v6, associated with highly invasive breast cancer and poor outcome [39].

This hypothesis was confirmed by Q-PCR which showed a significantly increased expression of twist1, (fold change 4,99 in KBr1 and 2,16 in KBr2) and snail1 (fold change 7,55 in KBR1 and 4,46 in KBr2) in both breast cancer samples, showing higher migratory activity after co-culture with ASCs (Figure 2B). A not significant change in CD44v6 and c-Met expression was found. In contrast, expression of those genes was found unchanged in KBr3 and KBr4 cells, after co-culture with ASCs (Figure 2B), even if KBr4 showed a 2,23 fold increase in c-Met, it was not parallel with an increased protein levels (Figure 2C).

Even more so, co-cultured normal epithelial cells, which revealed unable of migratory activity, did not display a metastatic signature, showing unchanged expression of twist1, snail1, CD44v6 and c-Met (Supporting Information, Figure 3C).

**Figure 3:**
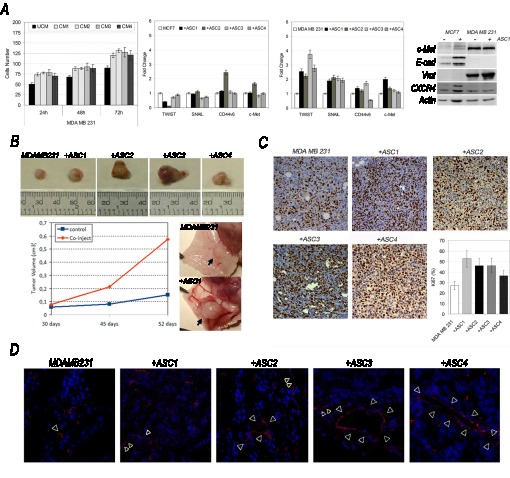
ASCs influence behaviour of MDA-MB-231 cells A) Invasion assay (ASCs-conditioned (CM) versus unconditioned (UCM) medium), metastatic signature and c-Met, Vmt, E-cad and CXCR4 protein levels in co-cultured MCF7 or MDA-MB-231 cells (+ASCs) versus MCF7 and MDA-MB-231 grown alone. B) Tumorigenicity, vascularization and C) Ki67 Evaluation, D) CD31 immunostaining (red, white-arrowheads, nuclei counterstained with DAPI) in co-injected (+ASCs) or single-injected (MDA-MB-231) xenografts.

Acquisition of a metastatic signature, associated to increased migratory activity in KBr1 and KBr2 co-cultured with ASC1 and ASC2, respectively, was not parallel with increased expression of cellular receptors involved with migration, as c-Met and CXCR4, or with higher vimentin and lower E-cadherin levels, proteins regulating cellular adhesion (Figure 2C), although western blot revealed that primary breast cancer cells, which demonstrated susceptible to ASCs during co-culture (KBr1 and KBr2), displayed higher levels of c-Met in comparison with KBr3 and KBr4, whose behaviour in culture appeared not influenced by the presence of ASCs.

### ASCs influence aggressiveness of tumorigenic primary breast cancer cells in vivo

Mesenchymal stem cells are known to be dispersed into the stroma of human tissue, having a role in tissue regeneration and homeostasis. Then, we wondered if co-injection of ASCs and breast cancer cells into immuno-compromised mice could influence breast cancer cells behaviour in terms of tumorigenicity and acquisition of a more aggressive phenotype in vivo. To address that issue we performed subcutaneous co-injections of ASCs with normal or cancer cells from mammary tissues.

We found that KBr1 and KBr2 cells generated tumors increased in size after co-injection, in comparison with tumors arisen when breast cancer cells were injected alone (Figure 2D and Figure 2E, KBr1 and KBr2 respectively). However ASCs did not affected behaviour of KBr3 and KBr4 breast cancer cells, which demonstrated to be not tumorigenic in mice, even when co-injected with ASCs. Besides, ASCs failed to induce a tumorigenic transformation in normal epithelial cells after co-injection.

Immunohistochemistry analysis of xenografts tissue sections revelead a significant increased Ki67 value in tumors generated by co-injection of ASCs with KBr1 (40%) or with KBr2 (30%) in comparison with control tumors which showed the same value of primary breast cancer tissues (20% and 10% respectively) (Figure 2D and E).

In order to evaluate if co-injection of ASCs induced phenotypic modifications of breast cancer cells, we evaluted the expression of hormonal receptors (ER, PR and Her2) in xenografts origining from breast cancer cells injected alone or in combination with ASCs (Additional file, Figure 4) and compared with expression found in primary tumors as well (Additional file, Table 1). No change was found in hormonal receptor status, which resembled the phenotype observed in primary tumor (Additional file, Figure 4).

Interestingly, CD31 immunostaining of xenografts tissues revealed that tumors, arisen after co-injection, showed a broad vascularization, increased in number and size of blood vessels (Figure 2D and E, co-inj) in comparison with tumors arisen after injection of primary breast cancer cells alone, (Figure 2D, cntr).

### ASCs influence proliferation, invasivity and tumorigenic behaviour of MDA-MB-231 cells

To evaluate if breast cancer phenotype could be relevant for breast cancer cells susceptibility to ASCs, we performed co-culture experiments in vitro using two different breast cancer cell lines: MCF7, with a luminal-like phenotype, and MDA-MB-231, with a basal-like phenotype.

We combined each of breast cancer cell lines above mentioned with four different ASCs (ASC1, ASC2, ASC3, ASC4) and evaluated in vitro and in vivo interactions.

Proliferation assay revealed an increase in proliferation rate when epithelial cells were co-cultured with ASCs (Additional file, Figure 2B), although a change in malignant phenotype in vitro was seen only in MDA-MB-231 cells.

Indeed, we found MDA-MB-231 increased their migratory activity when stimulated with ASCs-conditioned medium (Figure 3A), in contrast MCF7 remained unable to migrate, even in presence of ASCs-conditioned medium.

Migratory capabilities of co-cultured MDA-MB-231 cells were supported by increased expression of twist1 and snail1 genes (Figure 3A) and vimentin protein. Noticeably, western blot analysis revealed MDA-MB-231 cells expressed higher levels of c-Met in comparison with MCF7 (Figure 3A). No significant change was found in E-cadherin and CXCR4 after co-culture in both cells lines, except for a modest increase of vimentin in co-cultured MDA-MB-231 cells (Figure 3A).

To evaluate if tumorigenic behaviour of MCF7 and MDA-MB-231 could be susceptible to ASCs in vivo, we co-injected both cell lines into immunocompromised mice, alone or in combination with ASCs from four different donors. We found ASCs exacerbated tumorigenic potential of MDA-MB-231 cells, making them able to form tumors increased in size (Figure 3B) showing a higher Ki67 value (Figure 3C).

In addition, at the time of tumor resection from mice, we found xenografts appeared higly vascularized (Figure 3B), as confirmed by CD31 immunostaining (Figure 3D) revealing the presence of larger blood vessels in tumors origining after co-injection.

ASCs failed to induce a phenotypic transformation in MCF7 which did not reveal a tumorigenic and more aggressive behaviour in presence of ASCs, in vitro or in vivo.

### ASCs collaborate with breast cancer cells to sustain tumor angiogenesis

In vivo experiments performed using breast cancer cells, both primary cultures (KBr1 and KBr2) and cell lines (MDA-MB-231), revealed that co-injected ASCs could sustain tumor angiogenesis.

Contrasting evidences were reported, some of which suggesting that ASCs could differentiate into endothelial-like cells when recruited to the site of tumor formation [40]. Other evidences sustain undifferentiated cancer cells, maybe CICs, differentiate into endothelial cells [41].

To shed light on the mechanisms governing tumor-angiogenesis, we evaluated if breast cancer cells were able to recruit ASCs inducing their differentiation into endothelial-like structures.

**Figure 4:**
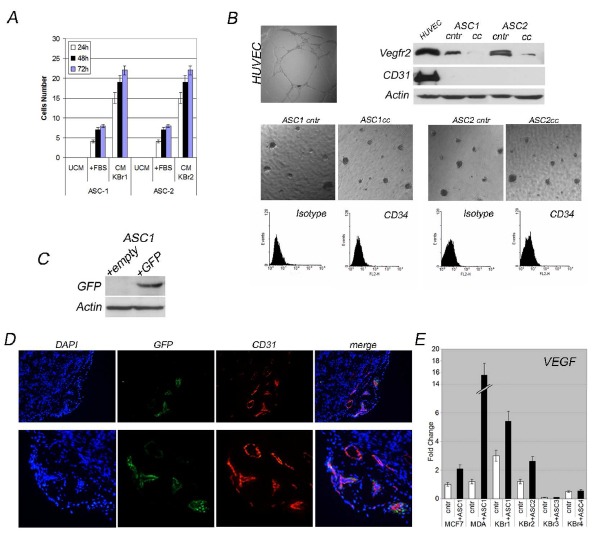
ASCs sustain tumor angiogenesis A) ASCs migratory activity. B) Angiogenic potential of co-cultured ASCs. HUVEC cells as control. Evaluation of VEGFR2, CD31 expression and CD34+ subpopulation in co-cultured ASCs versus ASCs grown alone. C) GFP expression in ASC1 trans-infected cells. D) Murine CD31 (red) in xenografts from co-transplanted MDA-MB-231 and GFP-expressing ASC1 (green). Nuclei counterstained with DAPI. D) VEGFR expression in breast cancer cells co-cultured versus grown alone.

A migration assay revealed that those cells were able to migrate when stimulated with conditioned media from primary breast cancer cells (Figure 4A), as well as a wound healing assay established both ASCs and primary breast cancer cells, moved toward each other showing a scatter phenotype, frequently depicted as associated to c-Met activation, completely covering the free area after five days (Additional file, Figure 2D).

Moreover hematoxylin/eosin staining after 5 days revealed the presence of two morphologically different cellular types, one displaying a fibroblast-like morphology, likely ASCs cells, and another with a small rounded morphology, likely breast cancer cells (Additional file, Figure 2D, H&E). Noticeably, H&E staining did not suggest the presence of vessel-like structure as result of ASCs differentiation, rather displayed ASCs surrounding breast cancer cells as stromal cells in breast cancer tissues.

This evidence was further supported by a transwell assay allowing us to study behaviour of ASCs in matrigel when co-cultured with primary breast cancer cells. We found that co-cultured ASCs were unable to form vessels, indeed they displayed unable to form vessels even when cultured alone in endothelial-specific medium, in contrast with endothelial cells (HUVEC) which formed a broad vasculature (Figure 4B).

In addition, cytofluorimetry analysis did not revealed an increased CD34+ subpopulation after co-culture, as well as western blot did not display increased expression of VEGFR2 or CD31 in co-cultured ASCs (Figure 4B), all surface markers for endothelial cells.

Altogether, those findings ruled out that ASCs could differentiate into endothelial cells in vitro, under stimulation of breast cancer cells, rather more likely ASCs could recruit endothelial progenitors cells from blood circulation to the site of tumor.

To verify this hypotesis we co-injected MDA-MB-231 cells with GFP-expressing ASCs (Figure 4C, western blot) into nude mice and evaluated contribution to tumor-angiogenesis in xenografts by murine endothelial progenitors cells. CD31 immunostaining of xenografts carried out using an antibody specific for murine CD31 (unable to recognize human CD31) showed a broad extensive vessels network supporting murine cells involvement into tumor-angiogenesis, besides no merge between GFP-positive and CD31-positive cells was found, rather GFP-positive cells localized next to endothelial cells (Figure 4D) as pericytes do, supporting endothelial vasculature.

Recruited progenitor endothelial cells localized into the tumor where high amount of VEGF were produced by cancer cells (MDA-MB-231, KBr1 and KBr2), rather ASCs which produced undetectable levels of VEGF as revealed by Q-PCR (Figure 4E), promoting their differentiation into endothelial cells.

### Increased HGF expression correlates with the ability of ASCs to influence tumorigenic behaviour of epithelial cells

Mesenchymal stem cells are known to produce several cytokines and chemokines essential in tissue homeostasis. Those diffusible factors act in a paracrine fashion making ASCs able to communicate with other surronding cell types. Considering that, we wondered if a crosstalk between ASCs and breast cancer cells could realize and why not all breast cancer cells are responsive to this communication.

Multiplex cytokine Array was performed in order to evaluate which, among ASC-specific cytokines and chemokines, underwent a significant increase when ASCs were co-cultured in presence of breast cancer cells.

**Figure 5:**
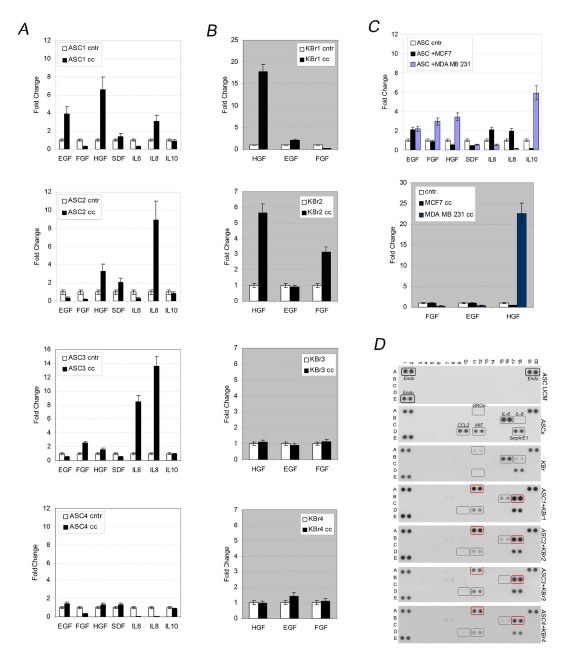
Co-culture increases HGF expression A) Cytokines and growth factors in co-cultured ASCs (cc) versus ASCs (cntr). B) Growth factors in KBr, co-cultured (cc) or grown alone (cntr). C) Cytokines and growth factors in ASC1 co-cultured with MCF7 or MDA-MB-231 versus ASC1 grown alone (cntr) (upper graph). Growth factors in MCF7 or MDA-MB-231 co-cultured with ASC1 (cc) or alone (cntr) (bottom graph). D) Cytokines/Chemokines profiles in ASCs or KBr cells grown alone or in co-culture condition.

We found that ASCs expressed detectable levels of several cytokines and chemokines as IL6, CCL2, MIF and Serpin-E1 (Figure 5D, ASCs) moreover we found Gro-alpha and IL8 production increased after co-culture, in contrast IL6, CCL2 and MIF levels significantly reduced (Figure 5D, ASC1+ KBr1, ASC2+KBr2, ASC3+ KBr3, ASC4+ KBr4). Noticeably, among those cytokines GRO-alpha and IL6 were produced at detectable levels even by primary breast cancer cells alone (Figure 5D, KBr).

Despite those changes, no correlation was found between cytokines/chemokines production and changes in behaviour of primary breast cancer cells grown under influence of ASCs, since all the ASCs-KBr combinations displayed similar cytokines/chemokines profiles.

To focus on cytokines and growth factors specifically produced by ASCs (EGF, FGF, HGF, SDF, IL6, IL8, IL10) or epithelial breast cancer cells (EGF, FGF, HGF) known to be relevant in tumorigenesis, we carried out Q-PCR analyses both on ASCs or primary breast cancer cells, before and after co-culture.

We found both ASC-specific or breast cancer cells–specific diffusible factors changed their expression after co-culture (Figure 5A and Figure 5B), however only HGF expression significantly correlated with the ability of ASCs to influence tumorigenic behaviour of epithelial. Indeed, HGF levels were very high both in ASC1 and ASC2 (Figure 5A, respectively 6,7 and 3,8 fold change), and KBr1, KBr2 cells (Figure 5B, respectively 16 and 6 fold change) which demonstrated exacerbated aggressiveness after combination. In contrast HGF expression was significantly lower (Figure 5A, respectively 1,5 and 1,2 fold changes) in ASCs co-cultured with KBr3 or KBr4. Noticeably, those epithelial breast cancer cells, whose behaviour remained unchanged in presence of ASCs, displayed HGF levels similar to control cells (Figure 5B).

Those findings were further confirmed by experiments with MCF7 and MDA-MB-231 cells, showing that HGF expression increased when ASCs were co-cultured with c-Met-expressing MDA-MB-231 cells (Figure 5C, upper graph, 3,8 fold change), which increased their own HGF production in turn (Figure 5C, lower graph, 22 fold change). HGF levels remained unchanged after co-culture in MCF, as expected considering MCF7 were not susceptible to ASCs (Figure 5C, upper and lower graphs).

### HGF/c-Met pathway mediates a crosstalk between ASCs and breast cancer cells

Observation that HGF production increased in co-cultured ASCs is parallel with the previous observation that breast cancer cells susceptible to ASCs expressed higher levels of c-Met (Figure 2C), suggesting a master role for HGF/c-Met pathway in crosstalk between ASCs and breast cancer cells.

In order to investigate about the master role HGF/c-Met pathway could have in crosstalk between ASCs and breast cancer cells favouring tumorigenesis, we repeated co-culture experiments in vitro, inhibiting c-Met in epithelial cells by simultaneous treatment with a specific inhibitor of its kinase activity.

**Figure 6:**
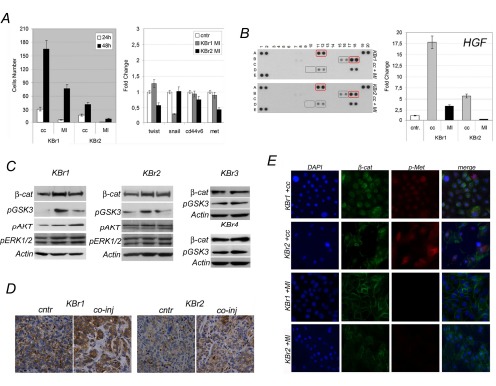
HGF/c-Met mediate crosstalk between ASCs and cancer cells A) Migratory activity and metastatic signature, B) Cytokines profiles and HGF expression and C) Signaling proteins, in co-cultured KBr (cc) with or without c-Met inhibitor (+MI). D) beta-catenin localization in xenografts from co-transplanted (co-inj) versus single-injected KBr cells (cntr). E) beta-catenin and phospho-c-Met in co-cultured KBr with (+MI) or without (+cc) c-Met inhibitor. Nuclei counterstained with DAPI.

We found c-Met inhibition did not change proliferation rate of co-cultured breast cancer cells, KBr1 and KBr2, which was similar to controls (Additional file, Figure 2C), in contrast significantly halted migration capacity of both KBr1 and KBr2 in co-culture with ASCs (Figure 6A, left graph), which expressed twist1 and snail1 levels similar to controls cells (Figure 6A, right graph).

Multiplex Cytokines Array revealed c-Met inhibitor treatment did not interfere with pro-inflammatory cytokines and chemokines produced by co-cultured ASCs (Figure 6B), which still displayed the same cytokines/chemokines profiles, however a dramatic reduction of HGF was highlighted by Q-PCR in both co-cultured KBr1 and KBr2 (Figure 6B).

To shed light on the mechanisms accounting for breast cancer susceptibility to ASCs, we evaluated the involvement of main pathways by western blot.

We found KBr1 and KBr2 displayed high levels of pAKT and pErk1/2, and a slight increase in pAKT was found after co-culture with ASCs, suggesting proliferation could be sustained by activation of AKT pathway, (Figure 6C, cc lanes), although it seems not dependent on c-Met activation, since c-Met inhibitor revealed unable to reduce pAKT levels (Figure 6C, +MI lanes).

Interestingly we found that breast cancer cells expressed increased levels of beta-catenin and phosphorylated GSK3 after co-culture with ASCs, suggesting HGF/cMet crosstalk could induce beta-catenin activation by GSK3 inhibition (Figure 6C, cc). Moreover, c-Met inhibition halted both beta-catenin stabilization and phosphorylated GSK3 levels (Figure 6C, +MI) suggesting beta-catenin stabilization, could rely on c-Met activation likely via GSK3 phosphorylation. Indeed, we did not found beta-catenin stabilization and increased GSK3 phosphorylation in normal breast cells (Additional file, Figure 3D) as well as in co-cultured KBr3 and KBr4 (Figure 6C, cc).

In contrast, a slight phospho-AKT increase in KBr4 as well as very high levels shown in KBr3, could explain the gain in proliferation rates (Additional file, Figure 2A) in presence of ASCs, although it was not sufficient to promote tumor growth and aggressiveness, suggesting low c-Met expressing cells as cancer cells with a low tumorigenic behaviour (Figure 6C).

Implication of beta-catenin to mediate HGF/c-Met signals was further confirmed by beta-catenin mislocation in xenografts generated after co-injection of breast cancer cells, KBr1 and KBr2, with ASCs (Figure 6D). In particular, we found that beta-catenin lost its cortical localization accumulating into cytoplasm, likely moving to cell nucleus.

Moreover, we found beta-catenin mislocation was associated to c-Met activation by Tyr1234/Tyr1235 phosphorylation. In vitro experiments point out nuclear accumulation of beta-catenin in co-cultured KBr1 and KBr2 cells also displaying phosphorylated c-Met (pMet) (Figure 6E, KBr1 cc and KBr2 cc). Interfering with c-Met activation during co-culture impaired beta-catenin nuclear translocation in both breast cancer cells, which returned to display a cortical beta-catenin (Figure 6E, KBr1 +MI and KBr2 +MI).

### HGF/c-Met mediated crosstalk between ASCs and breast cancer cells controls tumor self-renewal potential

Experiments performed using c-Met inhibitor suggested HGF/c-Met crosstalk between ASCs and breast cancer cells could have an important role in promoting cancer cells migration and tumor growth, making breast cancer cells highly invasive and successful in metastatic dissemination. Moreover, it revealed able to regulate beta-catenin localization suggesting an additional role of the HGF/c-Met mediated crosstalk in tumor self-renewal, via regulation of cells subpopulation endowed with stem-like properties.

To verify this hypothesis, we interfered with c-Met expression, inducing c-Met overexpression in MCF7 or c-Met silencing in MDA-MB-231 and evaluated in vitro and in vivo effects of co-culture with ASCs.

**Figure 7:**
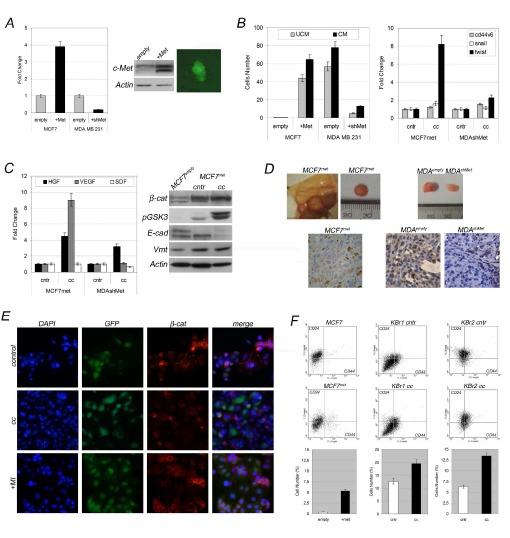
c-Met expression in cancer cells predicts ASCs-susceptibility A) c-Met expression in MCF7^met^ and MDA-MB-231^shMet^ cells versus control cells infected with empty vector (respectively MCF7^empty^ and MDA-MB-231^empty^). c-Met expression and anchorage-independent growth in MCF7^met^ (green) versus MCF7^empty^ (cntr). B) Migratory activity, metastatic signature and C) growth factors expression in co-cultured versus grown alone MCF7^met^ and MDA-MB-231^shMet^. beta-catenin/GSK3 interplay evaluation and EMT markers in co-cultured MCF7^met^ versus MCF7^met^ grown alone or MCF7^empty^. D) Tumorigenicity of co-injected MCF7^met^, MDA-MB-231^shMet^, MDA-MB-231^empty^ with ASCs and following beta-catenin immunostaining in vivo . E) beta-catenin (red) and GFP (green) immunostaining in *in vitro* co-cultured MCF7^met^ with (+MI) or without (cc) c-Met inhibitor versus MCF7^empty^ cells. F) CD44^+^/CD24^−/low^ subpopulation in co-cultured MCF7^met^, KBr1 and KBr2 cells versus un-cocultured (cntr) cells.

We found c-Met overexpression stimulated adhesion-independent growth in MCF7 cells (Figure 7A) which lost luminal features (lower E-cadherin, higher vimentin, Figure 7C), acquiring migratory capabilities (Figure 7B, UCM) previously not shown by MCF7 cells, in particular migratory capability was further stimulated in presence of ASCs (Figure 7B, CM) which promoted acquisition of a metastatic signature as suggested by increased snail1 and twist1 expression (Figure 7B,right panel, control represented by MCF7^met^ cells cultured alone).

Q-PCR revealed that MCF7^met^ produced higher amount of HGF and VEGF when co-cultured with ASCs, in contrast SDF1 expression was unchanged (Figure 7C, cc). In addition, no significant change was seen in cytokines/chemokines profile in comparison with MCF7^met^ cultured alone, still supporting that ASCs did not undergo a change in their pro-inflammatory activity, when co-cultured with phenotypically different breast cancer cells.

Basically, induced c-Met overexpression was sufficient to promote acquisition of an aggressive phenotype, dictated by migratory capabilities, metastatic signature, anchorage-independent growth, previously not showed by MCF7 cells, making those cells susceptibile to ASCs which in turns revealed able to exacerbate in vitro behaviour and tumorigenicity in mice (Figure 7D).

Conversely after c-Met silencing, MDA-MB-231 lost their susceptibility to ASCs, which demonstrated unable to sustain tumor growth and aggressiveness when co-injected with MDA-MB-231^shMet^ (Fig.7D).

Moreover, it was further confirmed beta-catenin involvement to mediate HGF/c-Met signaling, in breast cancer cells and ASCs crosstalk. Western blot confirmed beta-catenin stabilization via GSK3 phosphorylation (Figure 7C) and beta-catenin immunostaining revealed protein mislocation in MCF^met^ cells (green, because they express GFP) (Figure 7E). Effects on beta-catenin in vitro and in vivo, were severely impaired in primary breast cancer cells and in MCF7^met^ cells treated with c-Met kinase inhibitor (Figure 6E and Figure 7E) as well as in xenografts origining from MDA-MB-231^shMet^ (Figure 7D).

beta-catenin could explain the acquisition of a more aggressive, highly invasive, phenotype, upon c-Met activation, likely controlling the amount of cancer cells showing stem-like properties, reported to be associated with acquired chemoresistance, spreading and higher metastatic potential [42, 21].

Indeed, cytofluorimetric analysis revealed increased percentage of CD44^+^/CD24^−/low^ cells in MCF7^met^ cells and co-cultured breast cancer cells as well (KBr1 and KBr2) (Figure 7F, cytofluorimetric panels and bottom graphs), which was parallel to acquired adhesion-independent cells growth in KBr1 and KBr2 co-cultured in presence of ASCs (Additional file, Figure 2E). Besides, c-Met inhibitor severely impaired sphere forming (Additional file, Figure 2E, +MI).

### A positive staining for c-Met in primary tumors could predict susceptibility of breast cancer cells to ASCs

**Figure 8:**
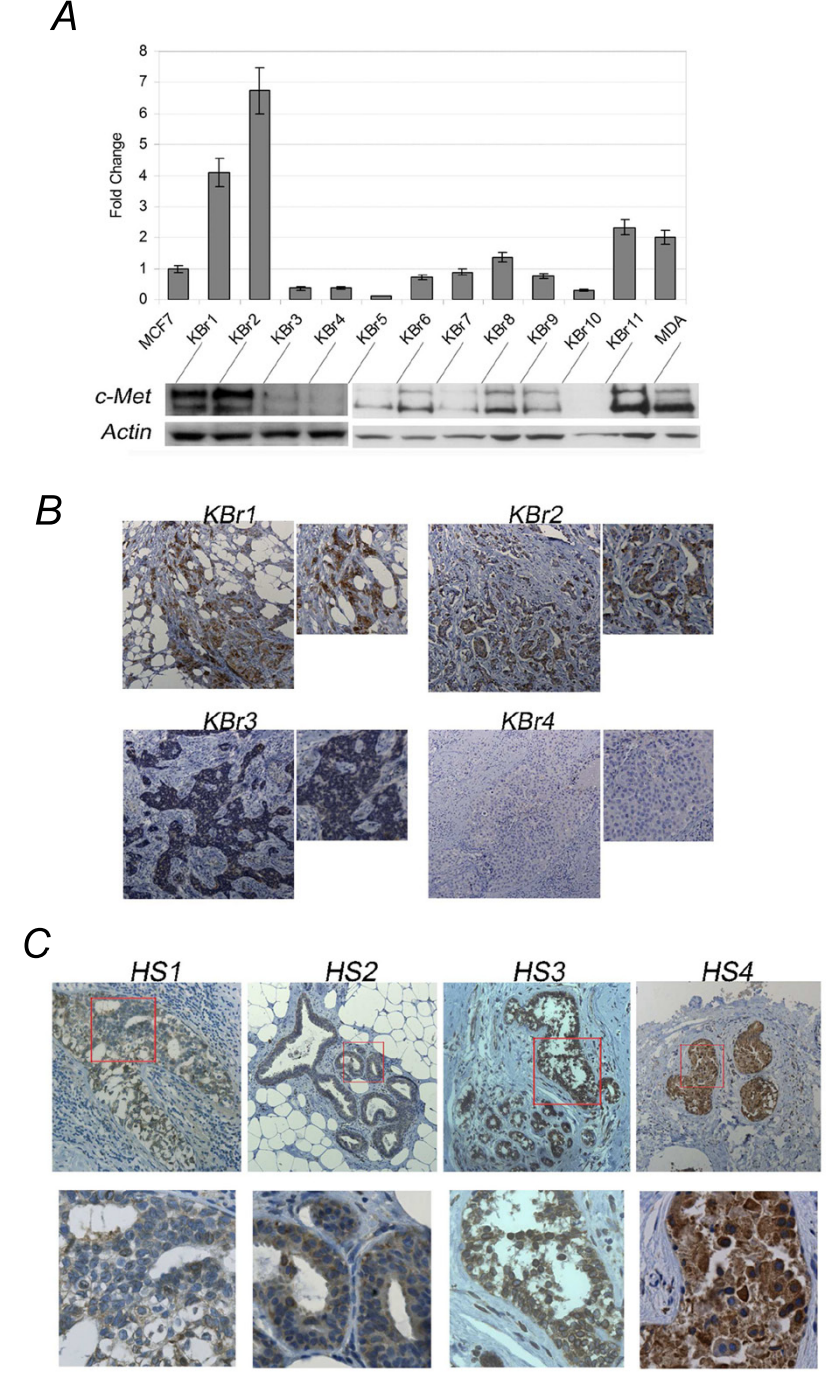
c-Met predicts recurrence after lipo-mediated breast reconstruction. A) c-Met expression and B) immunostaining in breast cancer tissues and in C) primary breast cancers of human donors showing (HS3 and HS4) or lacking (HS1 and HS2) recurrence following fat grafts.

To evaluate the role c-Met expression could have in predicting susceptibility of breast cancer cells to ASCs, we evaluated expression levels of c-Met in primary breast cancer cells isolated from eleven human donors which were co-injected with autologous ASCs into immuno-compromised mice to assess tumorigenicity. We found three (KBr1, KBr2, KBr11) out eleven samples revealed exhacerbated tumorigenicity which correlated with higher expression levels of c-Met in comparison with MDA-MB-231, as assessed by Q-PCR and Western blot (Figure 8A). In contrast, the remaining eight samples which displayed a low tumorigenic potential and revealed not susceptible to ASCs, expressed lower levels of c-Met.

Moreover, we carried out c-Met immunostaining in primary breast tumors of KBr1, KBr2, KBr3, KBr4 human donors, revealing a strong c-Met staining in KBr1 and KBr2, in contrast with weak staining in KBr3 and KBr4 (Figure 8B).

If c-Met could be a predictive marker for breast cancer patients at major risk of recurrence, when subjected to breast reconstruction by fat graft, we should find higher c-Met positivity in breast cancer patients which showed a recurrent pathology after fat grafts.

Indeed, c-Met immunostaining in four breast cancer samples (Additional file, Table 2) revealed a strong positivity in two samples orgining from patients displaying a recurrent pathology after 4 and 7 months from the first fat graft, a weak/moderate staining was seen in two samples origining from patients without signs of recurrence after 6 and 22 months from the first fat graft (Figure 8C).

## DISCUSSION

The interaction between epithelial and stromal cells plays a fundamental role in human tissues development and homeoastasis, insuring proper tissue regeneration after injury. However, recently growing evidences support the point of view assessing that mesenchymal stem cells could sustain cancer cells growth, confusing tumor for a “wound never healing”[43].

Based upon this evidence we investigated the role that ASCs could have in recurrence of breast cancer patients, undergoing autologous fat graft for breast reconstruction. Our aim was to dissect signaling pathways which could sustain a crosstalk between primary breast cancer cells and ASCs, favouring tumorigenesis.

We established a model where autologous ASCs and primary breast cancer cells from human donors were combined in order to evaluate potential effects of their interactions, in vitro and in vivo.

In vitro, we found co-cultured breast cancer cells displayed a gain in proliferation rate and invasive capabilities, acquiring a metastatic signature (Figure 2). In vivo, simultaneous co-injection of primary breast cancer cells and ASCs into nude mice suggested ASCs did not differentiate into adipocytes (Additional file, Figure 2, H&E), rather integrated into the tumor stroma, exacerbating tumorigenicity of primary breast cancer cells, which formed tumors increased in size and highly vascularized, although breast cancer cells phenotype, in terms of hormonal receptors, remained unchanged (Additional file, Figure 4).

However our findings revealed that ASCs were not tumorigenic per sè, since they were not able to induce a neoplastic transformation in normal mammary cells, which failed to form tumors in vivo (Additional file, Figure 3).

Surprisingly, we found that ASCs were not able to influence the behaviour of all primary breast cancer cells: namely, KBr1 and KBr2 revealed ASC-susceptible, exacerbating their tumorigenic capabilities both in vitro and in vivo, in contrast KBr3 and KBr4 behaviour appeared unchanged in presence of ASCs (Figure 2).

This observation was supported by experiments carried out with breast cancer cell lines, MCF7 and MDA-MB-231. When combined with the above-mentioned ASCs samples, MDA-MB-231 revealed increased aggressiveness in each combination (Figure 3), even when combined with ASC3 and ASC4 which previously revealed unable to influence tumorigenic behavior of KBr3 and KBr4. In contrast, MCF7 still revealed not tumorigenic, even when combined with ASC1 and ASC2 which previously revealed able to increase aggressiveness of KBr1 and KBr2 (Figure 3).

Our findings raised the question may rather breast cancer cells to influence ASCs behaviour in order to sustain their own growth and transformation, a guarantee for tumor development and metastatic spread.

Indeed, we found breast cancer cells, which revealed susceptible to ASCs (KBr1, KBr2 and MDA-MB-231), expressed higher levels of c-Met in comparison with breast cancer cells whose behaviour did not change in presence of ASCs (KBr3, KBr4, MCF7) (Figure 2 and 8). Noticeably, we found c-Met expression of primary breast cancer cells was parallel with c-Met expression found in primary tumor counterparts (Figure 8B). Moreover, we tested eleven primary breast cancer cells for tumorigenic potential into nude mice, after co-injection with autologous ASCs. We found that three (KBr1, KBr2 and KBr11) samples, showing to be ASCs susceptible, expressed higher levels of c-Met (Figure 8A).

Our data pointed out to a crosstalk between ASCs and breast cancer cells mediated by HGF/c-Met signaling.

Indeed, we found ASCs increased HGF production in presence of c-Met positive primary breast cancer cells (Figure 5A), which increased their own HGF production in turns (Figure 5B).

ASCs are well known to produce cytokines and chemokines important for inflammatory response to accomplish with a physiological role in tissue homeostasis, however we found they did not change cytokines/chemokines profiles under influence of breast cancer cells (Figure 5A).

This observation suggested that inflammatory potential of ASCs is not determinant per sè, although it could create a microenvironment where cancer cells are continuously stimulated to proliferate. HGF, produced and released into tumor microenvironment by both ASCs and breast cancer cells, could have a dual role: on the one hand, it could favor recruitment of several cell types to tumor sites, on the other hand, acting as a transforming factor for c-Met expressing breast cancer cells, could exacerbate tumor phenotype by stimulation of migratory activity and acquisition of a metastatic signature, giving tumor a chance to spread in different tissues.

Actually, HGF/c-Met was reported to be required for mammalian gland development [44], and was found deregulated in several human cancers, accounting for increased invasiveness and tumorigenicity [45] predicting poor outcome in breast cancer patients [39].

All primary breast cancer cells showed a gain on proliferation in vitro and markedly in vivo, in presence of ASCs, maybe due to cytokines/chemokines produced and inflammatory response generated, however it revealed not sufficient to sustain tumor growth in vivo, as we showed that low tumorigenic primary breast cancer cells still failed to generate tumors.

This suggest that a well orchestrated action between ASCs and primary breast cancer cells is required. Indeed we showed, both ASCs and tumorigenic breast cancer cells collaborating in tumor-angiogenesis, the former recruiting endothelial progenitors cells from blood to tumor sites (Figure 5C) and the latter releasing high amount of VEGF (Figure 4E) which promoted their differentiation into new blood vessels. Moreover, ASCs act as pericytes (Figure 4D), known to take part into endothelial cells maturation and functions, and increase survival of new growing vessels, which will supply nutrients and oxygen necessary to sustain tumor growth [45].

Dissecting the pathways to explain tumorigenic role of ASCs in vitro and in vivo, we found an increased expression of pERK1/2 and pAKT in co-cultured breast cancer cells that could drive ASCs-mediated cell proliferation.

Those kinases may represent good candidates for targeted therapies. Several reports showed that tumor microenvironment may influence chemoresistance of cancer cells through dis-regulation of AKT/mTOR pathway. Indeed, Rapamycin treatments [47, 48] revealed very efficient in the treatment of those cancers where tumor microenvironment has a leading role in tumorigenesis; however it could reveal unable to interfere with tumorigenic behaviour of ASCs shown in our results. In fact, experiments carried with c-Met inhibitor suggest that activation of pERK1/2 and pAKT could be independent on c-Met activation, since interfering with its kinase activity did not influence cell growth (Figure 6C). Moreover a gain in proliferation was also reported in normal mammary cells (N-Br) or in KBr3 and KBr4 after co-culture with ASCs, however it did not correlate with neoplastic transformation.

Noticeably, inhibiting c-Met kinase we were able to interfere with migratory activity and acquisition of metastatic signature of co-cultured breast cancer cells (Figure 6A), and significantly reduce HGF expression, as well (Figure 6B).

These findings highlight a dual role of HGF, released by both ASCs and breast cancer cells: First it acts as a chemoattractant to recruit different cell types (epithelial cells, endothelial progenitors, fibroblasts and immune cells), creating an inflammatory microenviroment, which sustains cancer cells growth. In addition, it favours tumor-angiogenesis induced by recruitment of endothelial progenitors into a microenvironment where breast cancer cells release high amount of VEGF (Figure 4E). In addition, cytokines/chemokines-mediated recruitment of fibroblasts and immune-cells contributes to the maintenance of inflammation, providing a further support to cancer cells proliferation.

Moreover our findings reveal an additional role of HGF/c-Met signaling in co-cultured breast cancer cells: the regulation of CICs subpopulation via beta-catenin regulation.

Indeed, we found that c-Met activation induced beta-catenin stabilization in breast cancer cells, mediated by GSK3 inactivation after phosphorylation, likely promoted via PI3K as previously reported [48]. Inhibition of c-Met impaired beta-catenin stabilization, reducing phopsho-GSK3 levels (Figure 6C).

Besides, we found beta-catenin mislocation from cell cortex, accumulating into the cytoplasm and/or into the nucleus, in vitro and in vivo (Figure 6D and E), effect impaired by c-Met inhibitor.

Nuclear traslocation of beta-catenin may explain metastatic signature acquired by co-cultured breast cancer cells, since it was reported to regulate twist1 expression [50], moreover suggest HGF/c-Met activation could regulate the amount of cancer cells showing stem-like properties. Indeed, we found CD44^+^/CD24^−/low^ subpopulation of breast cancer cells increased in presence of ASCs (Figure 7F), suggesting HGF/c-Met mediated crosstalk could exacerbate tumorigenic potential and aggressiveness of tumor cells, increasing the amount of those cells displaying stem like features, well known to be associated with acquired chemoresistance, tumor spreading and higher metastatic potential [42, 21] accounting for tumor recurrence.

Our findings revealed a master role for HGF/c-Met crosstalk in mediating a tumorigenic role of ASCs in breast cancer, making c-Met a good target for developing new cancer therapies [51-54].

However, it should be consider that ASCs support breast cancer tumorigenesis through a well orchestrated mechanism involving different compartments of a pathological microenvironment [55], therefore combined therapies may reveal more effective to increase drug responsiveness of tumor cells [26, 27] as combining c-Met inhibitors with rapamycin treatment, blocking AKT-mediated cell proliferation, or with PI3K inhibitors impairing beta-catenin stabilization and tumor self-renewal, or with drugs specifically targeting signaling pathways sustaining CICs.

Moreover, we found that the HGF/c-Met crosstalk, maybe directly or through co-receptor Nrp1 [56], is important for tumor angiogenesis, acting in concert with VEGF released by breast cancer cells, therefore the combination of c-Met inhibitors and anti-angiogenic drugs could lead to a simultaneous targeting of c-Met and VEGF receptor increasing the effectiveness of chemotherapy.

The pivotal role of c-Met as a marker of breast cancer cells susceptibility to ASCs was further confirmed by induction of c-Met expression in MCF7 or c-Met silencing in MDA-MB-231 cells.

We found, c-Met overexpression was sufficient to make MCF7 cells susceptible to ASCs, acquiring an aggressive phenotype in terms of migratory capabilities, metastatic signature, anchorage-independent growth, increased CD44^+^/CD24^−/low^ subpopulation and tumorigenicity in mouse model (Figure 7).

Conversely, c-Met silencing in MDA-MB-231 cells, showed that c-Met inhibition in breast cancer cells severely impaired tumorigenic potential of ASCs (Fig.7 MDA-MB-231^shMet^).

Basically, inhibiting or inducing c-Met expression, we were able to control susceptibility of breast cancer cells to ASCs.

Altogether our findings raise some concerns in the employment of mesenchymal stem cells (as fat grafts) in cancer patients for regenerative purpose, namely in the setting of breast reconstruction after tumor resection by conservative surgery.

Although, there are relatively few experimental evidences providing a mechanistic description about the role of ASCs in regenerative therapy, several clinical reports sustain autologous fat grafts do not correlate with increased breast cancer recurrence rates [57, 58, 35].

However, those clinical studies lacked an appropriate control group, since recurrence is usually measured comparing two different cohort of patients, receiving or not receiving fat grafts after tumor resection, chosen on the basis of clinico-pathological features and not considering heterogenic variability among breast cancer patients, nowadays highlighted by identification of molecularly distinct profiles [59], moreover it seems to disregard that fat graft could not influence recurrence rates, rather aggressiveness of the recurrence itself, which could reappear with an exacerbated phenotype after fat graft, making tumor more difficult to treat.

Besides, conservative surgery is associated with higher recurrence rates in comparison with mastectomy, due to its purpose to eradicate breast tumor, preserving the major of the mammary gland. Indeed, it was reported to fail in removal of the overall tumor tissue in some cases of breast cancer displaying a diffusely infiltrative growth, as in Infiltrating Lobular Carcinoma, for which was reported 51% of positive margins after conservative breast cancer or in Intraductal Carcinoma, displaying 30% of locoregional recurrence versus 1% after mastectomy, or in Ductal Carcinoma in situ with microinvasion [60-62].

Actually, we found c-Met expression could predict potential ASCs susceptibility of breast cancer cells, since we found a strong c-Met positivity in breast cancers of human donors displaying a recurrent pathology after fat grafts and a weak/moderate staining in patients which did not display any sign of recurrence after fat grafts (Figure 8C).

In light of this work, if disseminated tumor cells could persist in mammary gland, they could support recurrence after fat grafts, under the influence of ASCs through the activation of HGF/c-Met/beta-catenin axis.

Even if further clinical confirmation are required, our work suggests c-Met as a marker to predict breast cancer recurrence risk after fat graft for breast reconstruction in post-surgery breast cancer patients. Nevertheless, it could reveal a marker for recurrence in all those cases in which ASCs are used with regenerative/reconstructive purpose in cancer patients.

## MATERIALS AND METHODS

### Ethics Statement

Investigation has been conducted in accordance with the ethical standards and according to the Declaration of Helsinki and according to national and international guidelines and has been approved by the authors' institutional review board.

### Cells Cultures

The employment of human specimens was approved by our institutional Central Ethic Committee (CEC) at “Salvatore Maugeri” Foundation. ASCs cells as normal (N-Breast) and cancer (K-Breast) primary cells were isolated from lipoaspirates or breast tissues, respectively, of human donors after informed consent has been obtained. Eligible patients from Breast Unit were identified as patients undergoing cancer resection and simultaneous remodeling of controlateral breast and willing to consider breast reconstruction by autologous fat grafts at a later stage.

For primary cells isolation, we proceeded as following. Briefly, lipoaspirates were treated with 1mg/ml collagenase (Invitrogen) for 1h at 37°C with gentle agitation and then centrifuged for 10 min at low speed. The cellular pellet was resuspended in alfaMEM (Euroclone) supplemented with 10% FBS, 100 units/ml penicillin and 0.1 mg/ml streptomycin (Euroclone Ltd UK).

For primary cells isolation, normal or cancer breast tissues were digested with 1mg/ml collagenase and 10ug/ml Hyaluronidase (Invitrogen) for 2h at 37°C with gentle agitation. Then centrifuged for 10 minutes at low speed. The cellular pellet was resuspended in Ham's/F12 medium (ratio 1:1) supplemented with with 5% FBS, 100 units/ml penicillin, 0.1 mg/ml streptomycin, 10ug/ml Insulin, 20ng/ml EGF (Euroclone Ltd UK).

Autologous ASCs and primary normal or breast cancer cells were indicated with the same progressive number and combined together for in vitro and in vivo experiments (ASC1 and KBr1, ASC2 and KBr2, ASC3 and KBr3, ASC4 and KBr4 ). We reported experimental evidences from normal epithelial mammary cells (NBr1) origining from a single patient. Breast cancer cell lines, MCF7 and MDA-MB-231 were as suggested by ATCC.

A transwell-mediated co-culture system was established in which epithelial cells and ASCs are allowed to interact by production of diffusible factors. Cells were seeded at 30% of confluency in each well of the system and monitored for one week, then processed for assays described in “results” section. Data are results of three independent experiments.

Inhibitor experiments was performed with 50 nM kinase II Met inhibitor (Calbiochem) for a period of 7 days. Fresh drug was added to culture medium every 48 hours.

### Differentiation Assay

Adipogenic differentiation was induced by culturing ASCs cells for 2 weeks in adipogenic medium (DMEM supplemented with 10% FBS, 200 μM indomethacin, 1 μg/mL insulin, 1 mM dexamethasone, 0.5 mM isobutylmethylxanthine (IBMX), 100 U/mL penicillin, 100 mg/mL streptomycin) and assessed using an Oil Red-O (Sigma-Aldrich) stain as an indicator of intracellular lipid accumulation.

Osteogenic differentiation was induced by culturing ASCs cells in osteogenic medium for 4 weeks (DMEM supplemented with 10% FBS 10 mM β-glycerophosphate, 0.15 mM ascorbate-2-phosphate, 10 nM 1,25-(OH) 2 vitamin D3, 10 nM dexamethasone, 100 U/mL penicillin, 100 mg/mL streptomycin) and assessed by staining with Alizarin S Red (Sigma-Aldrich).

Chondrogenic differentiation was induced after 4 weeks culture in chondrogenc medium (DMEM supplemented with 1% FBS, 6.25 μg/ml Insulin, 10ng/ml TGF-beta1, 50nM L-ascorbic-acid-2-phosphate). Differentiated chondrocytes were stained with Alcian blue 8GX (Sigma-Aldrich). Nuclei were counterstained with nuclear fast red (Lab Vision, Inc.).

Medium was replaced every 3 days for the duration of all the experiment. Differentiation assay was performed in all four ASCs samples isolated, pictures from ASC1 was reported, in example.

### Immunohistochemistry

Immunohistochemistry on human breast cancer tissues and tumor xenografts was performed on 5-μm-thick paraffin-embedded sections. Epitope retrieval was performed in pH6 Retrieval buffer (DAKO) in a warm bath, before incubation with rabbit polyclonal c-Met antibody (1:200, Santa Cruz), beta-catenin (1:100, Santa Cruz) Ki67 (DAKO) or isotype controls. LSAB®Plus/HRP kit from DAKO was used for HRP-Mediated antigene detection. For H&E staining, dewaxed sections were stained in hematoxylin for 5 minutes, washed in water, and then exposed for 1 minute to eosin. Immunostaining results were analysed by DM1000 Microscope (Leica) equipped with LAS (Leica) Software for image capture and analysis.

For Ki67 counting, at least ten randomly selected regions for slides were analyzed and a minimum of 500 nuclei was counted for each sample.

### Immunofluorescence

Immunofluorescence of tumor xenografts was performed on 5-μm-thick optimum cutting temperature–embedded cryosections, in primary normal or breast cancer cells, or ASCs.

Tissue sections were fixed in ice-cold acetone, whereas cells were fixed in 2% paraformaldehyde and permeabilized with 0.1% Triton-X100, then blocked with 1.5%BSA.

Samples were incubated ON at 4°C with rabbit polyclonal c-Met antibody (1:200, Santa Cruz), phospho-Met (Tyr1234/1235)(1:200, CST), CD31 (1:50 AbCam) mouse monoclonal antibody beta-catenin (1:100, Santa Cruz), GFP (1:400, Sigma-Aldrich), CK8 (1:100, Santa Cruz), Vimentin (1:100, Santa Cruz), E-cadherin (1:100, Santa Cruz) or isotype-matched controls. Then, cells were treated with FITC- conjucted anti-mouse (1:250, Sigma-Aldrich) or Cy5-conjucted anti-rabbit (Jackson) and counterstained with DAPI (Invitrogen).

Samples were analyzed on Leica DM1000 Microscope (Leica) equipped with LAS Software for image capture and analysis.

### Production of lentiviral particles and infection

Met cDNA was cloned into pCDH-CMV-MCS-EF1-copGFP lentiviral vector (System Biosciences), containing GFP (Green Fluorescence Protein) as reporter gene. pCDH-CMV-MCS-EF1-copGFP empty vector was used to generate GFP-expressing ASCs cells.

Lentiviral supernatants were collected 48h following transfection of the packaging HEK-293T cells using FuGENE® HD Transfection Reagent (Roche Applied Science).

3x10^5^MCF7 or ASCs cells was seeded and after 24 hours was exposed at 4 ml of lentiviral supernatant with the addition of 8 μg/mL polybrene (Sigma-Aldrich). Culture medium was replaced after 24 hours. Infection efficiency was evaluated counting GFP-positive cells. For c-Met silencing in MDA-MB-231 cells was carried out with PLK01 shRNA lentiviral vector (TRCN0000040044, Sigma-Aldrich) accordingly with manifacturer instructions.

### Flow Cytometry

Cells were cultured in control medium and harvested by tripsinization in blocking buffer solution (1.5% BSA solution) and incubated with the following fluorescein-conjucted antibodies: CD44, CD90, CD45 (Becton Dickinson) Epcam (DAKO), or stained with a phycoerythrin-conjugated CD133 (Miltenyi Biotech), CD24, CD34, CD117 (Becton Dickinson) as recommended by manufacturer. Appropriate fluorescein- or phycoerythrin-conjugated isotypes were used as control for each assay. Flow cytometric analysis used a flow cytometer (FACScalibur, Becton-Dickinson, San Jose, CA). Results were processed with CellQuest Software (Becton-Dickinson, San Jose, CA) for statistical analyses. Statistical evaluation of CD44^+^/CD24^low/−^ cells was obtained by three independent experiments. Standard deviations is indicated.

### MACS sorting

EpCAM positive cells from normal or tumor primary cultures, were positively selected by Magnetic-activated cell sorting (MACS, Miltenyi Biotec) using CD326 (EpCAM) microbeats and LS midiMACS colums (Miltenyi Biotec) in according to manufacturer instructions. Recovery of EpCAM+ cells was confirmed by cytofluorimetry.

### Proliferation Assay

Cell proliferation was evaluated on primary cancer cells cultured alone or with ASCs for 1 week. Viability was assessed using the CellTiter Aqueous Assay kit (Promega Corp.) according to the manufacturer's instructions. Proliferation rate was reported as results of three independent experiments. Standard deviation was indicated by error bars.

### Invasion Assay

1x10^4^ primary breast cancer cells or ASCs were plated on solidified growth factor–reduced Matrigel (BD) diluted 1:3 in serum-free medium in 8-μm pore size 24-well plate Transwell (Corning). Conditioned media from ASCs (ASC1, ASC2, ASC3, ASC4) were used as chemo-attractant for primary breast cancer cells (KBr1, KBr2, KBr3, KBr4, respectively); vice versa, conditioned media from epithelial cells were used as chemo-attractant for ASCs (in the same combination ASC-KBr). Chambers were incubated at 37°C for 1 week, results at 24-48-72 hours were reported. Unconditioned medium supplemented with 10% FBS was used as control to test intrinsic migratory capabilities of each sample.

Migrated cells were reported as average result of three independent experiments. Standard deviation was indicated by error bars.

### Sphere Forming Assay

Breast cancer cells and ASCs were suspended each at a density of 1x10^3^ cells/ml in Ham's/F12 supplemented with B-27® Serum-Free Supplement (Life technologies), and seeded into low adhesion 24-well plates (Corning). Sphere forming was monitored for 1 week and colonies were counted using an inverted light microscope DM5000B (Leica) equipped with a CCD camera and LAS software (Leica) for picture capture. Images were captured with 10x objective at room temperature. Experiments were repeated three times with three replicate in each experiment.

### Capillary tube formation on Matrigel matrix

3x10^4^ ASCs or HUVEC cells were suspended in ECM medium (Lonza) supplemented with 25 ng/ml bFGF and seeded into matrigel, previously allowed to gelify into 0,4-μm pore size Transwell 24-well culture plates. For co-culture experiments, 5x10^3^ breast cancer cells were seeded in the bottom well. Capillary tube formation was observed at different time points up to one week using an inverted light microscope DM5000B (Leica) equipped with a CCD camera and LAS (Leica) software for picture capture. Images were captured with 10x objective at room temperature.

### Real-Time PCR

Total RNA of each sample was retro-transcribed into cDNA using High Capacity cDNA Archive kit as recommended by manufacturer (Applied Biosystems). For amplification 50ng of cDNA/sample were used for each samples and plate reading performed by Vii7 Real-Time PCR systems (Applied Biosystems).

All amplification reactions were performed in triplicate, and the relative quantitation of genes expression was calculated using the comparative Ct method (DeltaDeltaCt). Glyceraldehyde-3-phosphate dehydrogenase (GAPDH) was used as endogenous control. Data processing and statistical analysis were performed using Vii7 software

Following the PCR reaction, a melting curve assay was performed to determine the purity of the amplified product when PCR was performed using Sybr Green chemistry.

### Western blot analysis

SDS PAGE was performed as previously described in [63]. Primary antibodies were: mouse monoclonal Actin (1:400), Vimentin (1:400), E-cadherin (1:100), beta-catenin (1:100)(Santa Cruz), pErk1/2 (1:400, Cell Signaling Tech), CXCR4 (1:200, ThermoFisher), VEGFR2 (1:100, Sigma-Aldrich) or rabbit polyclonal c-Met (1:200, Santa Cruz), pAKT (1:200) and pGSK3 (1:200) (Cell Signaling Tech.), CD31 (1:100, AbCam).

A representative picture of three independent experiments was reported. Western blots showed in Figure 2C were processed in parallel as western blot showed in Figure 8A.

### Cytokines Array panels

Co-culture with ASCs and primary breast cancer cells or breast cancer cell lines (MCF7 or MDA-MB-231) were performed in 6-well transwell assay. Conditioned medium from ASCs (2ml) was harvested after 7 days of culture. 700 μl of conditioned medium was assayed using the human cytokine array panel A (R&D systems) allowing to analyze 36 cytokines at a time, according to the manufacturer's instructions. A qualitative evaluation of cytokines/chemokines profiles was shown for co-culture ASCs. Conditioned media from ASCs and KBr grown alone, as well as culture medium (alphaMEM) were used as controls, one picture representative for each was shown.

### In vivo experiments

In vivo experiments were authorized by Italian Ministry of Health. Female Balb/c nude mice were obtained from the Harlan Laboratories and maintained according to national guidelines for animal care and use committee (Ministry of Health). For subcutaneous model were used the following cell lines: 5×10^6^ MCF7 and MCF7^met^, 3×10^6^ MDA-MB-231 cells; 1x10^6^ primary breast cancer cells (KBr1, KBr2, KBr3, KBr4 and NBr). All cell samples were suspended in 150μl PBS/Matrigel and were injected onto flank region of nude mice 4-5 weeks old. Primary breast cancer cells were injected alone or in combination with autologous ASCs in 2:1 ratio as for breast cancer cell lines.

Tumor mass size was measured weekly for up to 15 weeks (for injections with primary cells) or 8 weeks (for injections with cells lines), and volumes calculated according to the formula: (π/6)*larger diameter*(smaller diameter)^2^. Tumor growth data derive from six independent experiments.

## SUPPLEMENTARY FIGURES AND TABLES


