# Supplementary material for: Adipose-derived mesenchymal stem cells (ASCs) may favour breast cancer recurrence via HGF/c-Met signaling
Source: Oncotarget. 2013 Oct 23;5(3):613–33. doi: 10.18632/oncotarget.1359 (PMC3996669; doi:10.18632/oncotarget.1359)
Supplement: Supplementary file 1 [file oncotarget-05-613-s001.pdf]

Additional files:

-Figures Legends.

-Tables.

-Figures

Figures Legends.

Figure 1. **Characterization of primary breast cancer cells isolated from human donors.** Immunostaining for CK18, vmt , E-cad and β-cat. Evaluation of EpCam+ cells after sorting.

Figure 2. ASCs influence tumor cells behavior. A) Proliferation rates in co-cultured (co-culture) versus control (cntr) primary breast cancer cells B) and in co-cultured (+ASC1, +ASC2, +ASC3, +ASC4) MCF7 or MDAMB231 versus controls (cntr); C) or in co-cultured primary breast cancer cells with or without c-Met inhibitor (+MI). D) ASCs migratory activity. D) Wound healing assay seeding KBr (on the left) and ASCs (on the right). H&E staining 5 days after seeding. E) Sphere forming assay in co-cultured KBr1 and KBr2, with or without c-Met inhibitor (+MI) versus KBr1 and KBr2 grown alone.

Figure 3. Normal mammary epithelial cells are not susceptible to ASCs co-culture. A) Normal mammary cells morphology and markers (EpCam, CK18, E-cad). B) Proliferation rate C) Metastatic signature and D) Expression of pAKT, β-cat and pGSK3 in NBr1, cultured with (cc) or without (cntr) ASCs.

Figure 4. ASCs influenced breast cancer cells behaviour in vivo. Hormonal receptors (ER, PR, Her2) and H&E staining in xenografts from co-transplanted autologous ASCs and breast cancer cells or ASCs and $MCF7^{met}$.

Additional file Tables

Table 1. Hystopatological classification of tumor specimens.

Tumors	ER (%)	PR (%)	Her2	Ki67 (%)	Grading	Histotype
KBr1	80	70	1+	25	G2	IDC*
KBr2	80	40	0	10	G2	IDC*
KBr3	80	10	0	5	G2	IDC*
KBr4	60	70	3+	20	G2	IDC*

*IDC: Infiltrating Ductal Carcinoma.

Table 2. Hystopatological classification of tumor specimens.

Tumors	ER (%)	PR (%)	Her2	Ki67 (%)	Grading	Histotype	Recurrence
HS1	80	60	1+	8	G2	DIN2*	NO
HS2	80	70	0	20	G3	DIN2/3*	NO
HS3	0	0	3+	10	G3	DI+DIN3*	after 7 months
HS4	0	0	1+	10	G3	DIN3*	after 4 months

*DIN: Ductal Intraepithelial Neoplas

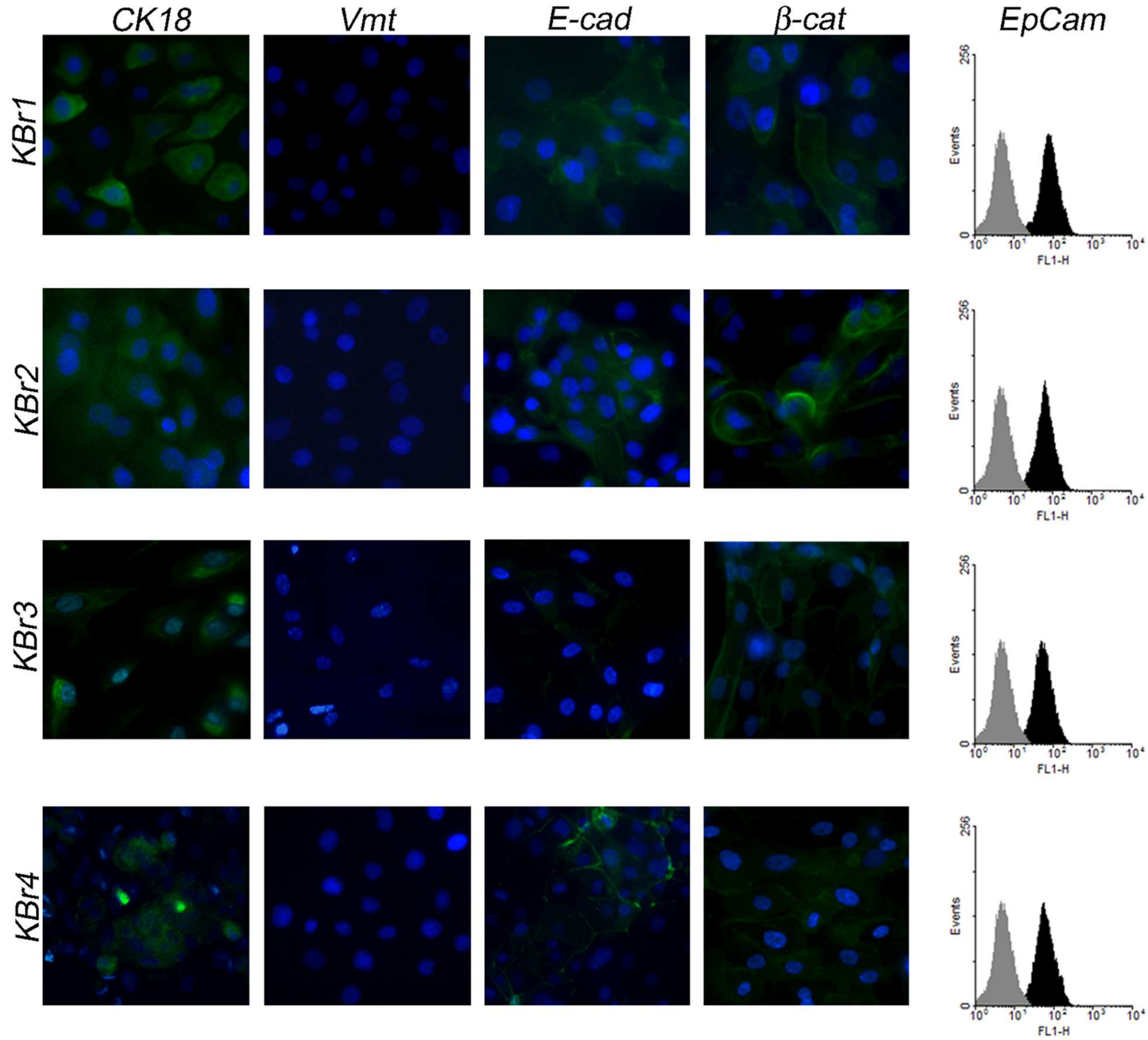

CK18
Vmt
E-cad
β-cat
EpCam
KBr1
KBr2
KBr3
KBr4
Events
FL1-H

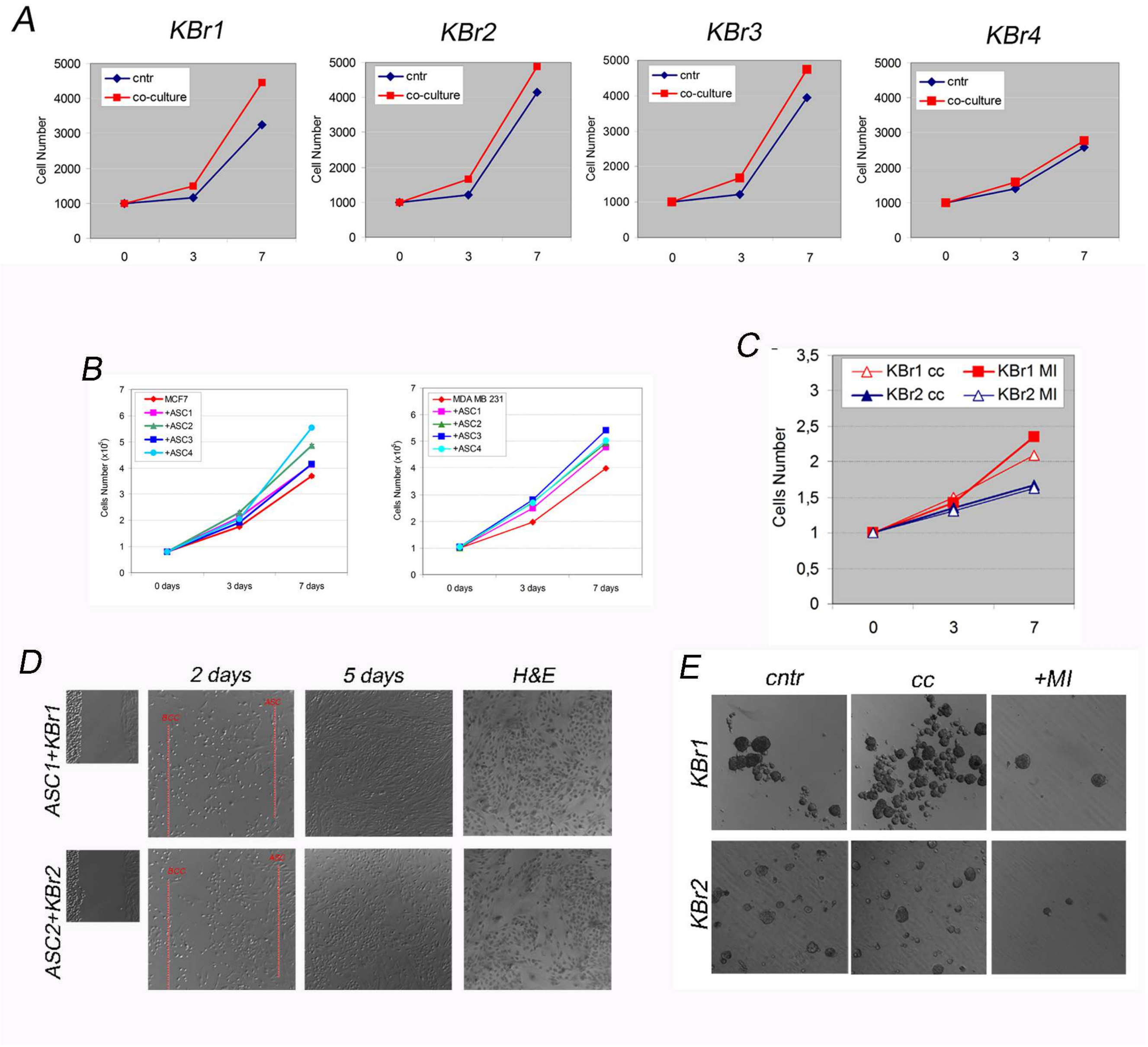

A
KBr1
KBr2
KBr3
KBr4
cntr
co-culture
Cell Number
0
1000
2000
3000
4000
5000
0
3
7
B
MCF7
+ASC1
+ASC2
+ASC3
+ASC4
MDA MB 231
Cells Number (x10⁵)
0 days
3 days
7 days
C
KBr1 cc
KBr1 MI
KBr2 cc
KBr2 MI
Cells Number
0
0,5
1
1,5
2
2,5
3
3,5
D
2 days
5 days
H&E
ASC1+KBr1
ASC2+KBr2
BCC
ASC
E
cntr
cc
+MI
KBr1
KBr2

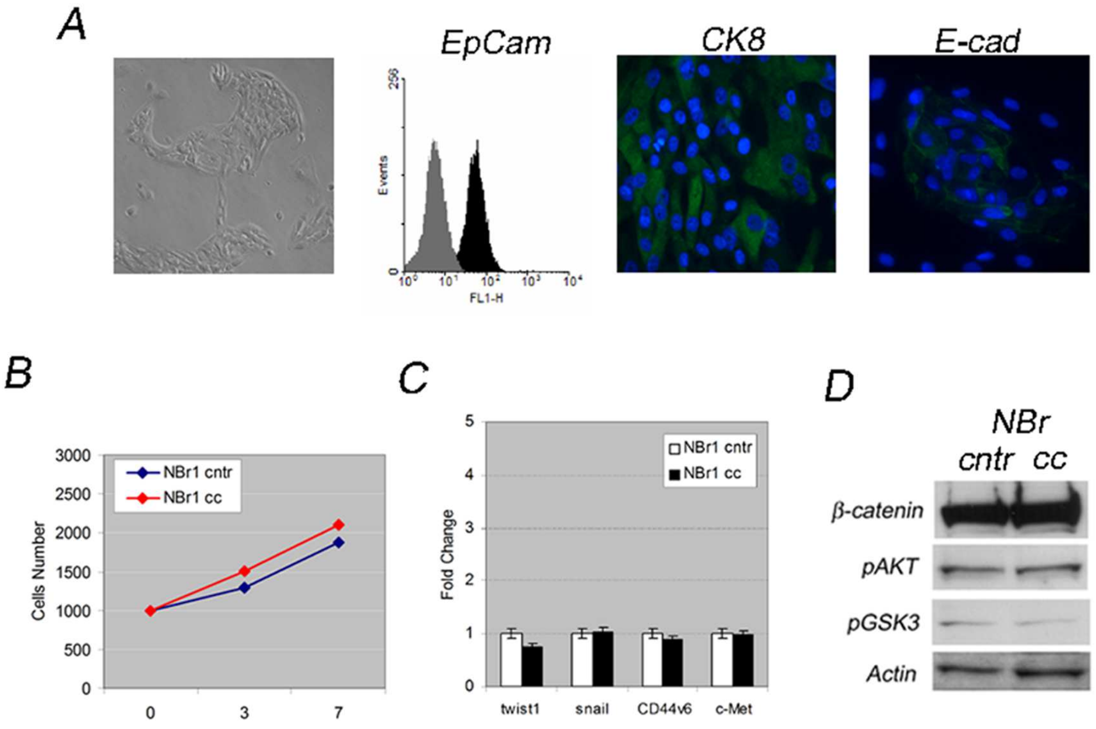
A
EpCam
CK8
E-cad
256
Events
0
FL1-H
B
NBr1 cntr
NBr1 cc
Cells Number
3000
2500
2000
1500
1000
500
0
0
3
7
C
NBr1 cntr
NBr1 cc
Fold Change
twist1
snail
CD44v6
c-Met
D
NBr
cntr
cc
β-catenin
pAKT
pGSK3
Actin

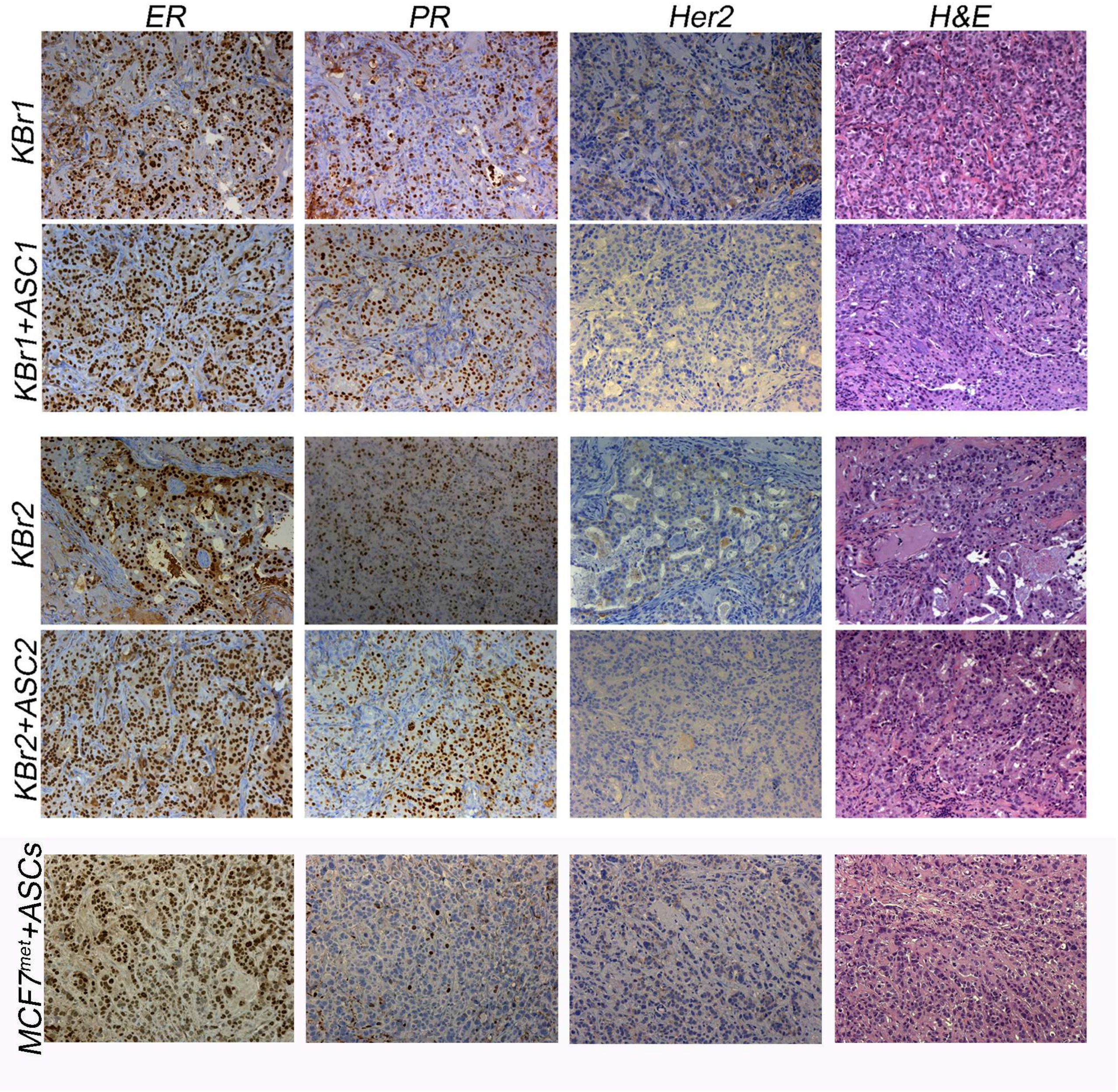

ER
PR
Her2
H&E
KBr1
KBr1+ASC1
KBr2
KBr2+ASC2
MCF7met+ASCs
